# STAT3 Facilitates Super Enhancer Formation to Promote Fibroblast‐To‐Myofibroblast Differentiation by the Analysis of ATAC‐Seq, RNA‐Seq and ChIP‐Seq

**DOI:** 10.1111/jcmm.70639

**Published:** 2025-06-04

**Authors:** Yujie Wang, Yaqin Zhao, Guohong Cao, Mengqi Jiang, Xinglong Yuan, Hongbo Li, Xiaodong Song, Jinjin Zhang, Changjun Lv, Songzi Zhang

**Affiliations:** ^1^ Department of Respiratory and Critical Care Medicine Binzhou Medical University Hospital, Binzhou Medical University Binzhou China; ^2^ Department of Cellular and Genetic Medicine Binzhou Medical University Yantai China; ^3^ Department of Medicine School of Medicine, CHA University Seongnam‐si Korea

**Keywords:** fibroblast‐to‐myofibroblast differentiation, H3K27ac modification, idiopathic pulmonary fibrosis, STAT3, super enhancer

## Abstract

A cellular characteristic of IPF is the transformation of fibrosis into myofibroblasts. This study identifies several transcription factors—STAT3, FOXP1, JUNB, ATF3, FosL2, BATF, Fra2 and AP‐1—that play crucial roles in promoting pulmonary fibrogenesis. They achieve this by facilitating the differentiation of fibroblasts into myofibroblasts, as analysed through ATAC‐seq and RNA‐seq. Additionally, STAT3 ChIP‐seq showed that STAT3 is significantly concentrated in accessible chromatin regions, including introns and intergenic areas. H3K27ac ChIP‐seq and Co‐IP demonstrated that STAT3 plays a role in the formation of super enhancer (SE), which promotes gene expression. CUT&RUN‐qPCR and the pGL3‐SE dual‐luciferase reporter system assays proved that STAT3 enhanced pGL3‐SE activities by facilitating H3K27ac modification, leading to promoting the transcription of target genes including RUNX1, JUNB, JUN, SMAD6, COL3A1 and PTPN1. In summary, this study shows that STAT3 contributes to the formation of SEs that accelerate the differentiation of fibroblasts into myofibroblasts, leading to IPF. This insight enhances our understanding of STAT3‐related SEs and offers potential therapeutic strategies for fibrotic diseases.

## 1 Background

Idiopathic pulmonary fibrosis (IPF) is an advancing and fatal lung disease with unknown causes, and there is currently no effective treatment available. The main features of IPF are a widened alveolar septum, damage to the alveolar structure, fusion of alveoli, and excessive connective tissue deposition. IPF progressively worsens dyspnea, ultimately resulting in hypoxemia and death from respiratory failure [1, 2]. A typical cellular characteristic of IPF is the differentiation of fibroblasts into myofibroblasts, followed by their proliferation and migration [3, 4, 5]. For example, the methyl‐CpG‐binding domain 2 facilitates pulmonary fibrosis by orchestrating fibroblast‐to‐myofibroblast differentiation [6]. *Sparganii Rhizoma* inhibits fibroblast differentiation to alleviate pulmonary fibrosis via the TGF‐β1/Smad2/3 pathway [7]. Recent advances in sequencing technology have significantly improved our understanding of epigenetic regulation, including chromatin accessibility and histone modification. However, the regulatory mechanisms behind activated fibroblast differentiation remain poorly understood. Therefore, it is crucial to study the epigenetic mechanisms of differentiation to establish a foundation for developing effective treatments for IPF.

Accessible chromatin refers to compact chromatin that has loosened, allowing DNA to be unpacked from nucleosomes and exposed to binding factors such as transcription factors (TFs), mediator proteins, DNA‐binding proteins and RNA polymerase. It is a key feature of active DNA regulatory elements that control physical access to nuclear DNA [8, 9, 10]. As a result, chromatin accessibility can indicate how TFs bind to DNA and the regulatory potential of genetic locations [11, 12]. Recent studies have shown that changes in chromatin accessibility play a role in cell differentiation, primarily through TF regulation and histone modification. Franklin et al. suggested that the commitment to specific cell fates is influenced by changes in chromatin structure and the activity of lineage‐specific TFs. They discovered that suppressing chromatin assembly factor‐1 (CAF‐1) triggers the rapid differentiation of myeloid stem and progenitor cells into a mixed lineage state; furthermore, they found that CAF‐1 sustains lineage fidelity by controlling chromatin accessibility at specific loci and limiting the binding of TF‐ELF1 at newly accessible diverging regulatory elements [13]. The TFs BTB and CNC homology 1 suppress chromatin accessibility at the promoters of the marker genes of vascular smooth muscle cells (VSMCs) by recruiting the histone methyltransferase G9a and cofactor YAP and maintaining the H3K9me2 state, thereby regulating the phenotypic transition of VSMCs [14]. Transposase‐accessible chromatin assays, known as ATAC‐seq, use an engineered hyperactive Tn5 transposase preloaded with sequencing adapters. This method is used to identify accessible chromatin sites. It provides a simple and scalable approach for assaying genomic regions that are bound by TFs and for comparing how these landscapes change in a particular context or under a certain perturbation [15, 16]. While chromatin accessibility likely drives the progression of pulmonary fibrosis, little is currently understood about this phenomenon.

Although ATAC‐seq is useful for comparing open chromatin regions in samples, it is often not used alone because it lacks quantitative interpretation. Moreover, given that gene expression depends on multiple regulatory regions, chromatin accessibility does not fully reflect gene expression [17, 18]. Combined analyses of ATAC‐seq, RNA‐seq and ChIP‐seq demonstrate that the accessible chromatin regions involved in transcription dysregulation differ between leukaemia cells and normal B‐cell progenitors [19]. The chromatin regulator bromodomain‐containing protein 8 (BRD8) preserves H2AZ occupancy at p53 target loci via the EP400 histone acetyltransferase complex. In TP53WT glioblastoma, targeting the bromodomain of BRD8 displaces H2AZ and enhances chromatin accessibility, which promotes p53 transactivation, leading to cell cycle arrest and tumour suppression [20]. The combined use of these sequencing technologies enhances our understanding of the regulatory mechanisms of epigenetics, genetics, and gene expression, including aspects like chromatin accessibility, histone modification, TF binding, and gene activation profiles.

In this study, we initially employed ATAC‐seq to assess changes in chromatin accessibility. Next, we identified key TFs that may play a role in fibroblast‐to‐myofibroblast differentiation by analysing TF‐binding motifs. Subsequently, we conducted RNA‐seq to identify differentially expressed genes. Based on the combined analysis of ATAC‐seq and RNA‐seq, we then performed ChIP‐seq to further investigate TF regulation and histone modification. In summary, this study offers a comprehensive overview of chromatin dynamics and TF motifs that regulate fibroblast differentiation. It also shows that STAT3 plays a crucial role in forming super enhancers, which promote the transition from fibroblasts to myofibroblasts, ultimately leading to pulmonary fibrogenesis.

## 2 Methods

### 2.1 Cell Model of Fibroblast‐To‐Myofibroblast Differentiation

Human fetal lung fibroblast MRC‐5 cells purchased from the American Type Culture Collection were cultured in minimal essential medium (MEM, Gibco), supplemented with 10% fetal bovine serum and 1% penicillin/streptomycin at 37°C in a 5% CO_2_ incubator. To establish the fibroblast‐to‐myofibroblast differentiation model, TGF‐β1 was administered at 5 ng/mL for either 6 or 72 h, based on the specific experimental requirements.

### 2.2 Western Blotting Detection

Cells were collected and lysed with a mixture of phenylmethylsulfonyl fluoride (PMSF) and radioimmunoprecipitation assay buffer. The protein concentration was measured by the Pierce BCA Pro kit. The protein samples were cooked at 95°C for 10 min in a boiler together with sample buffer. After being separated in sodium dodecyl sulfate polyacrylamide gel electrophoresis (SDS‐PAGE), the protein samples were transferred to polyvinylidene fluoride (PVDF) membranes. The membranes were washed three times with TBST buffer, then blocked with 5% nonfat dry milk for 2 h. They were then incubated overnight at 4°C with the following antibodies: anti‐collagen I (Affinity Bioscience, USA), anti‐collagen III (Affinity Bioscience, USA), anti‐vimentin (Affinity Bioscience, USA), anti‐α‐SMA (Affinity Bioscience, USA), anti‐FAP1 (Cell Signal Technology, USA), anti‐S100A4 (Cell Signal Technology, USA). The membranes were washed three times with 1× tris buffered saline Tween‐20 and then incubated with goat anti‐rabbit/mouse secondary antibody for 1 h. The expression of proteins was detected by enhanced chemiluminescence reagent kit (SparkJade, China).

### 2.3 qRT‐PCR Analysis

Cells were collected to extract RNA samples. Complementary DNA was synthesised with the Evo M‐MLV RT Premix Reagent (Accurate Biology, China) according to the manufacturer's instructions. The qRT‐PCR was performed on a Rotor Gene 3000 real‐time PCR system with the SYBR green PCR master mix kit (Takara, Japan). Amplification was performed as follows: 95°C for 30s, 40 cycles at 95°C for 5 s and 60°C for 30s, and a dissolution curve from 65°C to 95°C (+0.5°C one cycle).

### 2.4 Wound Healing Assay

MRC‐5 cells (5 × 10^4^/mL) were seeded into 96‐well plates and cultured at 37°C with 5% CO_2_. When the cell density reached 80%, the complete medium was replaced by serum‐free medium with or without TGF‐β1. Next, a line was drawn across the surface of cultured cells using the IncuCyte S3 scratcher (Sartorius, Michigan, USA). To remove dead cells, the cells were washed three times with 1 × PBS. After adding fresh medium, the cell samples were placed in the IncuCyte S3 live‐cell analysis system (Essen BioScience, USA) for real‐time monitoring of cell activity. The images were taken and analysed using the IncuCyte S3 software.

### 2.5 Cellular Proliferation Analysis

MRC‐5 cells were seeded in 6‐well plates at a concentration of 1 × 10^5^/mL. The samples were placed in the IncuCyte S3 live‐cell analysis system (Essen BioScience, USA) for real‐time monitoring of cell proliferation. Scheduled repeat scans were captured and analysed using the IncuCyte S3 software.

### 2.6 ATAC‐Seq Analysis

First, 50 μL of pre‐cooled resuspension buffer with 0.1% NP40, 0.1% Tween‐20 and 0.01% digitalis saponin was added to 5 × 10^4^ living cells, and the mixture was incubated on ice for 3 min. Next, 1 mL of pre‐cooled resuspension buffer containing only 0.1% Tween‐20 was added. The mixture was then centrifuged at 500 × *g* for 5 min. The supernatant was removed, and the precipitate was resuspended in 50 μL of transposition mix from the Nextera kit. This mixture was placed in a mixometer at 177 × *g* for 30 min. DNA purification was first performed using the Qiagen MinElute PCR Purification Kit, followed by a second purification with the Zymo DNA Clean and Concentrator‐5 Kit. Next, PCR was conducted in a final volume of 50 μL with the following steps: initial denaturation at 98°C for 1 min, followed by an extension at 72°C for 5 min. Seven cycles of PCR amplification were performed at 98°C for 15 s, 63°C for 15 s and 72°C for 1 min. The production of PCR was used for final library construction and high‐throughput sequencing on the Illumina HiSeq/NextSeq platform.

### 2.7 RNA‐Seq Analysis

We collected cells and extracted total RNA using TRIzol reagent, assessed its integrity and concentration with the Agilent 2100 RNA Nano 6000 Assay Kit (Agilent Technologies), constructed libraries following the manufacturer's instructions with the TruSeq Stranded mRNA LT Sample Prep Kit (Illumina, San Diego, CA, USA), and sequenced the cDNA libraries using 150 bp paired‐end sequencing on the Illumina HiSeq platform.

### 2.8 ChIP‐Seq Detection

Cell samples were crosslinked with 1% formaldehyde for 10 min at room temperature, after which glycine was added for 5 min to terminate the crosslinking. The cells were lysed, and the nuclei were sonicated to fragment the chromatin into pieces ranging from 200 to 500 base pairs. According to the manufacturer's instructions, antibodies including anti‐H3K27ac and anti‐STAT3 (both from Cell Signal Technology, USA) were added and incubated overnight at 4°C on a rotary mixer. DNA purification was performed using the DNA Clean & Concentrator‐5 Kit from Zymo Research. Sequencing libraries were prepared using the Next Ultra II DNA Library Preparation Kit from New England Biolabs (NEB).

### 2.9 Immunofluorescence Staining Observation

MRC‐5 cells were seeded on the cell climbing films in 24‐well plates. They were then fixed with ice‐cold absolute ethanol for 20 min and punched with 0.3% Triton X‐100. Cells were washed three times with PBS. They were blocked with goat serum for 30 min at 37°C, and then incubated overnight at 4°C with antibodies. After three washes with PBS, fluorescently labelled secondary antibodies were added and incubated at room temperature for 45 min, followed by another washing step. A volume of 200 μL of DAPI was added to each well to stain the nuclei for 6 min, followed by washing with PBS. Finally, the anti‐fluorescence quencher was applied to the cell slide, and all images were collected using a laser scanning confocal microscope (Zeiss LSM880, Germany).

### 2.10 Immunoprecipitation

MRC‐5 cells were collected and lysed for 10 min using pre‐chilled lysis buffer (Absinn, China) containing PMSF, and sonicated with a cell sonicator. The supernatant was collected by centrifugation at 4°C for 10 min at 14,000 × *g*, and then it was immunoprecipitated overnight at 4°C using either 5 μg of antibodies or normal rabbit IgG. Subsequently, 10 μL of protein A/G agarose beads (Absin, China) were added, and the mixture was gently stirred at 4°C for 3 h. The complexes bound to the protein A/G conjugate were washed. They were then resolved in SDS‐PAGE loading buffer and analysed using Western blotting.

### 2.11 Cleavage Under Targets and Release Using Nuclease (CUT&RUN) ‐qPCR

CUT&RUN was performed according to the manufacturer's instructions with the CUT&RUN Detection Kit (Vazyme, China). MRC‐5 cell samples were collected. Each reaction included 5 μL of either H3K27ac, IgG, or p‐STAT3 antibody for antibody binding at 4°C for 6 h. After washing three times, the cells were rotated and incubated with protein G‐fused micrococcal nuclease (pG‐MNase) at 4°C for one hour. Pre‐cooled CaCl_2_ was added to activate pG‐MNase, which released the antibody‐related protein‐DNA complex at 4°C for 90 min. Then, the stop solution was added to the reaction and incubated at 37°C for 20 min. The released DNA was purified by using the Fast Pure Gdna Mini Columns. Subsequently, qPCR was performed to detect target genes. The enrichment levels of H3K27ac and p‐STAT3 in the target genes associated with the super enhancer were assessed. Super enhancers (SEs) were identified using the ROSE algorithm [21], which ranks enhancers based on H3K27ac ChIP‐seq signal intensity and clusters adjacent enhancers within a 12.5 kb window. SEs were defined as the top‐ranked enhancer clusters exceeding this size threshold, as previously described. We normalised Spink DNA as an internal control and calculated the ratio relative to IgG in the negative control group for reference. The primers used for CUT&RUN‐qPCR were as follows: RUNX1‐F: CTTTCCTGGCTGCGAACTTT, RUNX1‐R: GTTACACACGCACGCACATC; JUN‐F: TGTTTCGGGAGTGTCCAGAG, JUN‐R: CGGGAAAGTTCTTTGCTGCT; JUNB‐F: ACATGGATGATGCTGGAAACC, JUNB‐R: AATGCTATCCCTCCCCTAGCC; COL3A1‐F: CTTGCTGTGGTGGTGTTGG, COL3A1‐R: CGGGGTTTTTACGAGAACCA; PTPN1‐F: TGGGGAGCCTAGATTCTGTGT, PTPN1‐R: CATGGGGCATTTTCTAGCAGT; SMAD6‐F: CAGCAGCAGGAAGGCATTT, SMAD6‐R: GCCCAGCTTTGTTTGCATT.

### 2.12 Animal Model and Ethical Statement

The animal experiments were conducted in accordance with the ethical guidelines established by the Animal Experiment Ethics Committee at Binzhou Medical University. Eight‐week‐old C57/BL6 mice, approximately 20 g in weight (±5 g), were obtained from Hangzhou Ziyuan Experimental Animal Science and Technology Company in Hangzhou, China. Based on the experimental requirements, the mice were randomly assigned to two groups of ten: the sham group and the BLM group. A Penn‐Century MicroSprayer (Penn‐Century Inc., Wyndmoor, PA, USA) was utilised to administer 5 mg/kg of BLM directly into the trachea. The sham group was sprayed with equal amounts of saline. On day 28, all mice were euthanized, and their lung tissue was collected and immediately frozen in liquid nitrogen for further analysis.

### 2.13 Statistical Analysis

Data were presented as means ± standard deviation (SD) and analysed using GraphPad Prism software. All statistical analyses were performed in GraphPad Prism using Student's t test for pairwise and ANOVA for multiple comparisons. Statistical significance was indicated as follows: *p ≤ 0.05; **p ≤ 0.01; ***p ≤ 0.001; ****p ≤ 0.0001; NS, not significant (*p* _ 0.05).

## 3 Results

### 3.1 ATAC‐Seq Reveals Changes in Chromatin Accessibility During Fibroblast‐To‐Myofibroblast Differentiation

**FIGURE 1:**
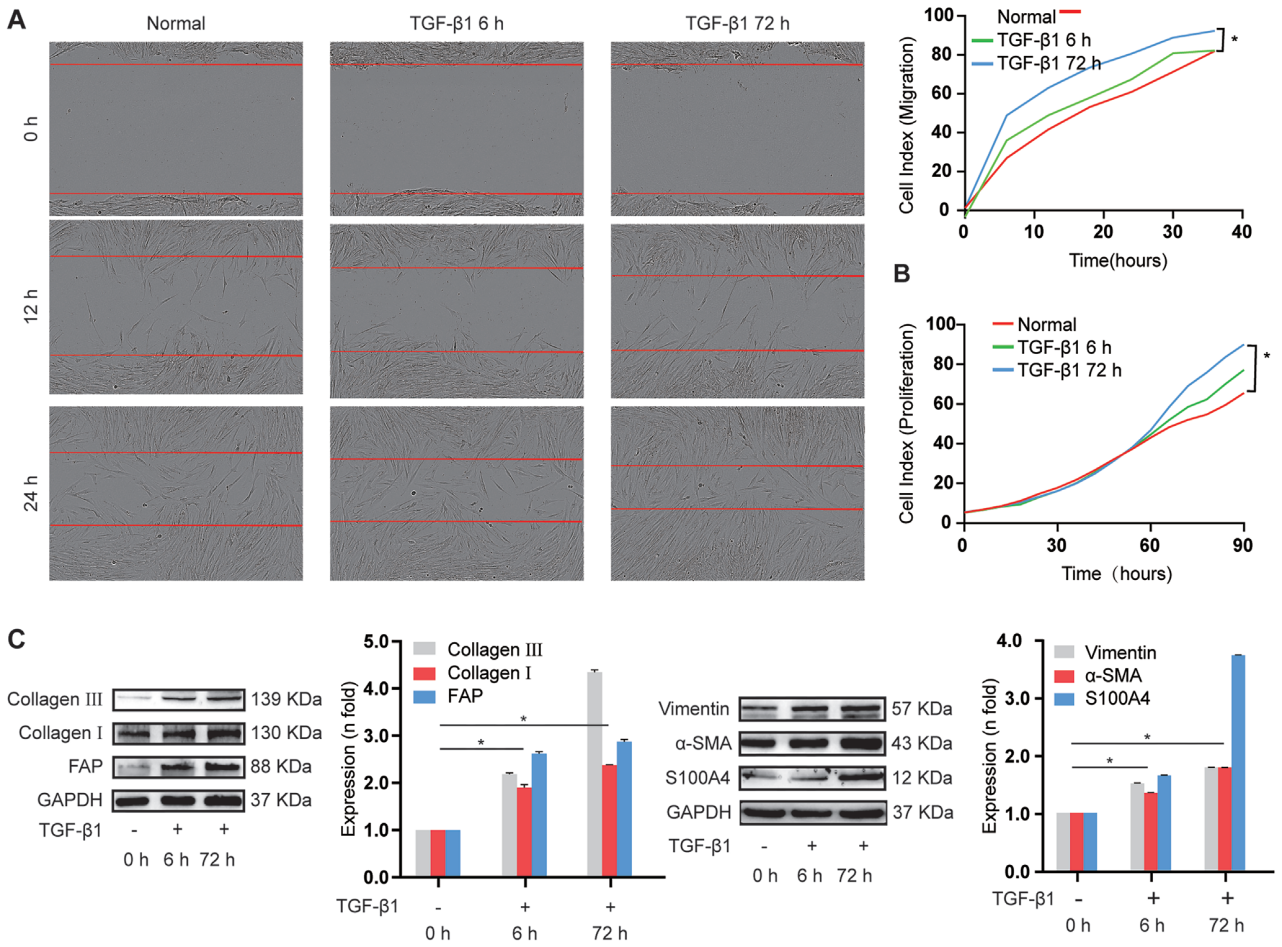
Establishment of the fibroblast‐to‐myofibroblast differentiation cell model. (A) Wound healing assay showed that the migration ability of MRC‐5 cells gradually increased under TGF‐β1 treatment. (B) IncuCyte S3 live‐cell analysis system depicted that the proliferation ability of MRC‐5 cells increased in the TGF‐β1‐stimulated group. (C) Western blot analysis illustrated that differentiation‐ and fibrosis‐related proteins were up‐regulated in TGF‐β1‐stimulated fibroblasts relative to in control cells.

A model of fibroblast‐to‐myofibroblast differentiation was established with 5 ng/mL TGF‐β1‐stimulated MRC‐5 cells for 6 and 72 h, as described in our previous studies [5, 22]. An IncuCyte S3 live‐cell analysis system and Western blotting analysis were used to evaluate the differentiation of fibroblast into myofibroblast, as well as their migration and proliferation. The scratch‐induced wounding assay showed that TGF‐β1‐treated cells were spindle‐shaped and exhibited increased migration and proliferation compared to control cells (Figure 1A,B). In TGF‐β1‐stimulated fibroblasts, we observed a high expression level of fibroblast activation protein (FAP), differentiation‐related protein S100 calcium‐binding protein A4 (S100A4), myofibroblast marker alpha sarcomeric actin (α‐SMA), and fibrosis‐related proteins including vimentin and collagen, compared to control cells (Figure 1C). These results suggest that the model of fibroblast‐to‐myofibroblast differentiation was successfully established.

**FIGURE 2:**
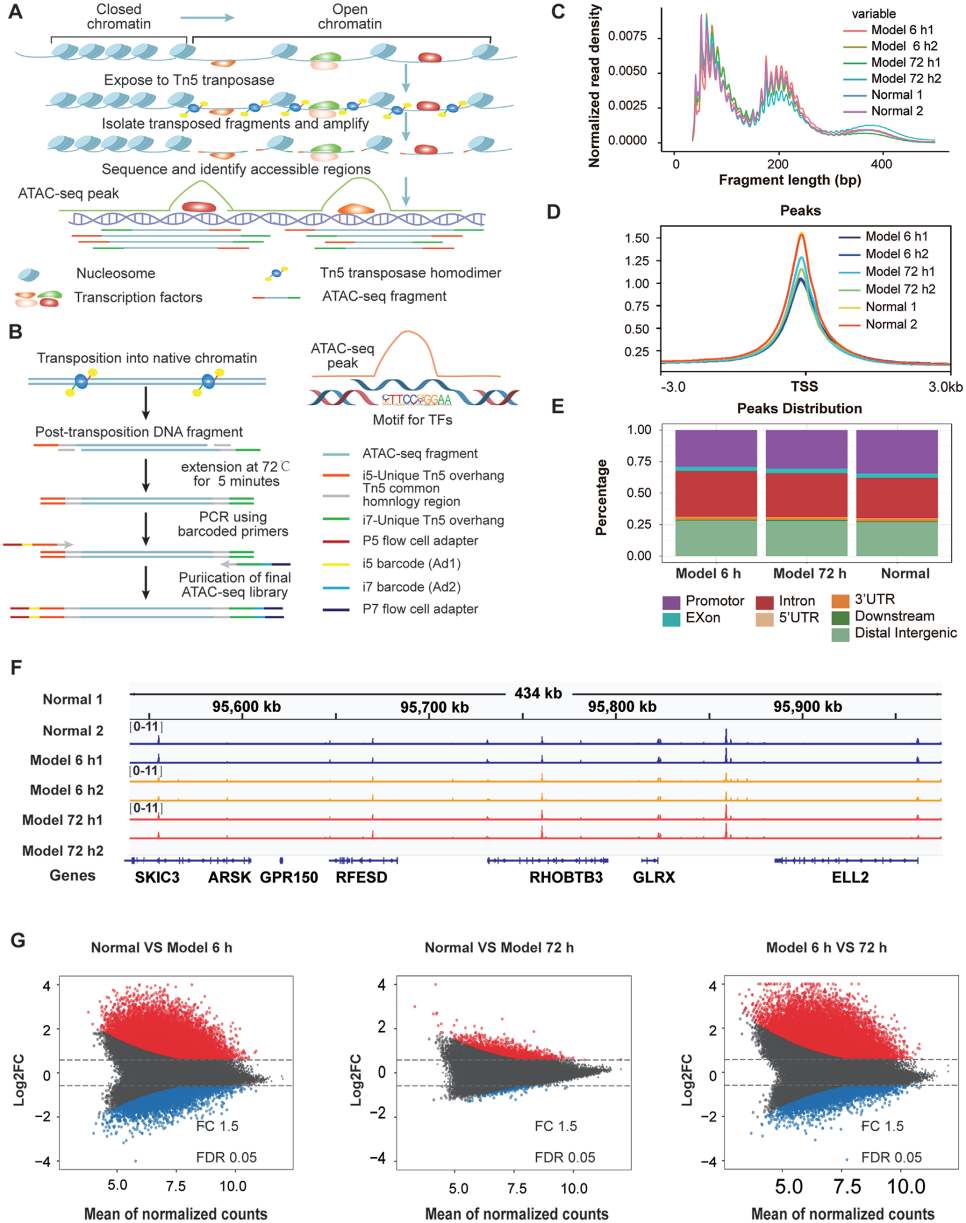
ATAC‐seq revealed alterations in chromatin accessibility profiles during fibroblast‐to‐myofibroblast differentiation. (A) Schematic of ATAC‐seq. Nuclei were isolated. Chromatin structure and DNA‐binding proteins, such as nucleosomes and TFs, remained intact. Subsequently, chromatin was exposed to Tn5 transposase and fragmented. The enrichment of Tn5‐accessible chromatin was designated as the ATAC‐seq peak. (B) Detailed schematic of DNA fragments resulting from transposability. Tn5 transposase–inserted and –fragmented chromatin. Only the fragments with ATAC‐seq adapters of i5/P5 at one end and i7/P7 at the other were properly amplified and sequenced. Before amplification, the adapters must complete the 72°C extension step. During subsequent PCR, fragments were labelled with common sequencing ends and barcodes. Homer2 tool was used to identify the TF‐binding motif associated with differential peaks. The STAT3 motif is an example of a TF‐binding motif. (C) Distribution of ATAC‐seq fragments with similar sizes in each group. (D) ATAC‐seq signals were enriched around TSSs. (E) Chart showing the genetic distribution of accessible chromatin regions, including exons, intergenic regions, introns, promoters, 3′UTRs and 5′UTRs. (F) IGV browser view showing the enrichment of ATAC‐seq peaks in the normal, 6 and 72 h model groups. (G) MA plot showing the changes in ATAC‐seq peaks in MRC‐5 cells with or without TGF‐β1 treatment. Red (up), blue (down), and grey (no change).

An ATAC‐seq library was constructed using MRC‐5 cells with or without TGF‐β1 treatment. Following library inspection, sequencing was conducted on an Illumina HiSeq X platform. Both raw reads and unique mapped reads were analysed. The Tn5‐accessible chromatin peaks indicate the enrichment of Tn5 transposition events in specific genomic regions of each sample, referred to as ATAC‐seq peaks. They contribute to screening the motif sequences of DNA‐binding TFs (Figure 2A,B). Quality control of the ATAC‐seq sequencing data showed that most inserted fragments were small (< 200 bp) with similar size distributions. This indicates that chromatin was accessible to Tn5 transposase and that regions of chromatin were open and not occupied by nucleosomes (Figure 2C). Meanwhile, ATAC‐seq signals were abundantly enriched at transcriptional start sites (TSS) (Figure 2D). Accessible peaks were annotated to explore the location of accessible chromatin in genes. Most of the accessible regions were located in promoter regions, then intergenic regions (distal intergenic) and intron regions (intron). Under TGF‐β1 stimulation, the proportion of accessible peaks in the promoter region decreased, while the proportion in intergenic and intron regions increased (Figure 2E). Furthermore, the ATAC‐seq results of each group of samples were visualised by using a randomised localised integrative genomics viewer (IGV). Sequencing data exhibited low noise and a significant peak, indicating good ATAC‐seq data quality (Figure 2F). Through peak calling and differential analysis in the model‐based analysis of ChIP‐seq 2, we quantitatively identified the regions of altered accessibility with the criteria of log2 fold change > 0.67 and false discovery rate < 0.05. The 6‐h model group displayed 21,448 and 16,929 regions of significant accessibility compared to the control group, while the 72‐h model group exhibited 2012 and 1870 such regions. In comparison to the 6‐h model group, the 72‐h model group had 18,128 and 10,307 significantly accessible regions (Figure 2G). The ATAC‐seq data indicated that fibroblasts undergo abnormal chromatin remodelling during their differentiation into myofibroblasts.

### 3.2 RNA‐Seq Identifies Differentially Expressed Genes Involved in Fibroblast‐To‐Myofibroblast Differentiation

**FIGURE 3:**
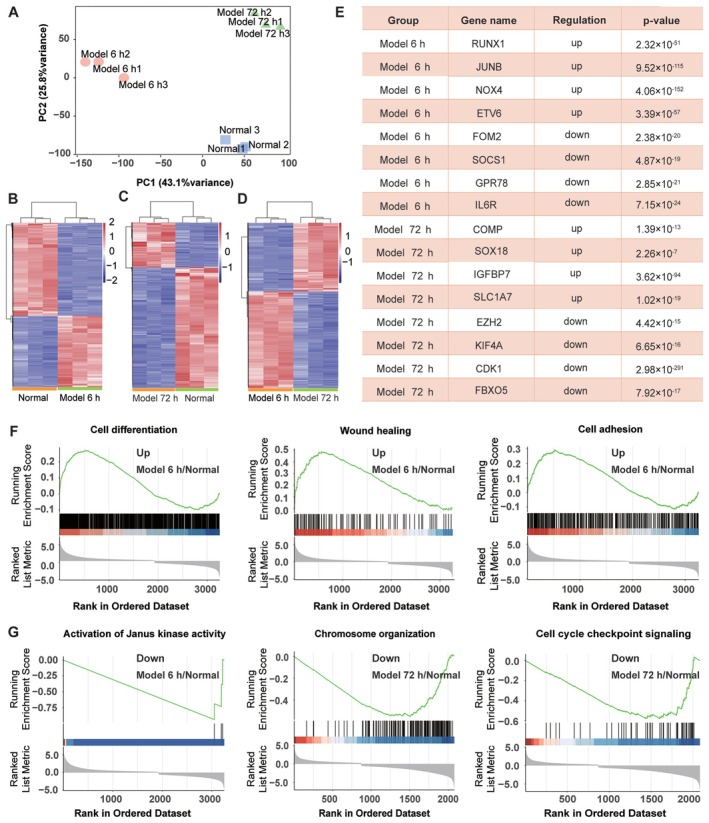
RNA‐seq revealed the differentially expressed genes during fibroblast‐to‐myofibroblast differentiation. (A) Principal component analysis scatter plot of gene expression profiles assessed by RNA‐seq. The percentage of variance accounted for by each principal component is indicated. (B–D) Heatmap of genes with altered expression in MRC‐5 cells under TGF‐β1 treatment for 6 or 72 h. (E) Representative up‐ and down‐regulated genes in the 6 and 72 h model groups relative to in the normal group. (F) GO biological processes associated with the up‐regulated genes in MRC‐5 cells with or without TGF‐β1 treatment for 6 h revealed by the GSEA algorithm. (G) GO biological processes associated with the down‐regulated genes in MRC‐5 cells with or without 6 or 72 h of TGF‐β1 treatment revealed by the GSEA algorithm.

Since chromatin accessibility is closely correlated with active transcription, we conducted RNA‐seq on the same samples batch used in the earlier ATAC‐seq analysis to investigate changes in gene transcription levels. After normalising the data and removing batch effects, we performed principal component analysis (PCA) to identify the primary sources of variation. We found that the control group and the TGF‐β1 treatment groups were significantly different from one another, while the samples within each group were homogeneous (Figure 3A). Hierarchical cluster analysis showed that 976 genes were up‐regulated and 1376 genes were down‐regulated in the 6‐h model compared to the control group (Figure 3B). Similarly, in the 72‐h model, 442 genes were up‐regulated and 1193 genes were down‐regulated (Figure 3C). Additionally, when comparing the 72‐h model to the 6‐h model, we found that 2159 genes were up‐regulated and 1659 genes were down‐regulated (Figure 3D). In the 6‐h model, the up‐regulated genes included RUNX1 and JUNB, while the down‐regulated genes included NUF2, EZH2 and KIF4A. In the 72‐h model, the up‐regulated genes were NOX4, ETV6, TEAD4 and FOXP1, and the down‐regulated genes were CDK1, SOCS1 and E2F1 (Figure 3E). Next, we performed gene set enrichment analysis (GSEA) to explore the biological processes associated with the differentially expressed genes, focusing on Gene Ontology Biological Process (GO‐BP) category [23]. The up‐regulated genes in the 6‐h model were significantly associated with cell differentiation, wound healing, and cell adhesion (Figure 3F), while the down‐regulated genes in the 6‐ and 72‐h models were significantly associated with Janus kinase activity, chromosome organisation, and cell cycle checkpoint signalling (Figure 3G). In line with previous studies [24, 25], our results showed that these biological processes were related to the development of pulmonary fibrosis.

### 3.3 Overview of Integrative Analysis Between Chromatin Accessibility and Gene Transcription

**FIGURE 4:**
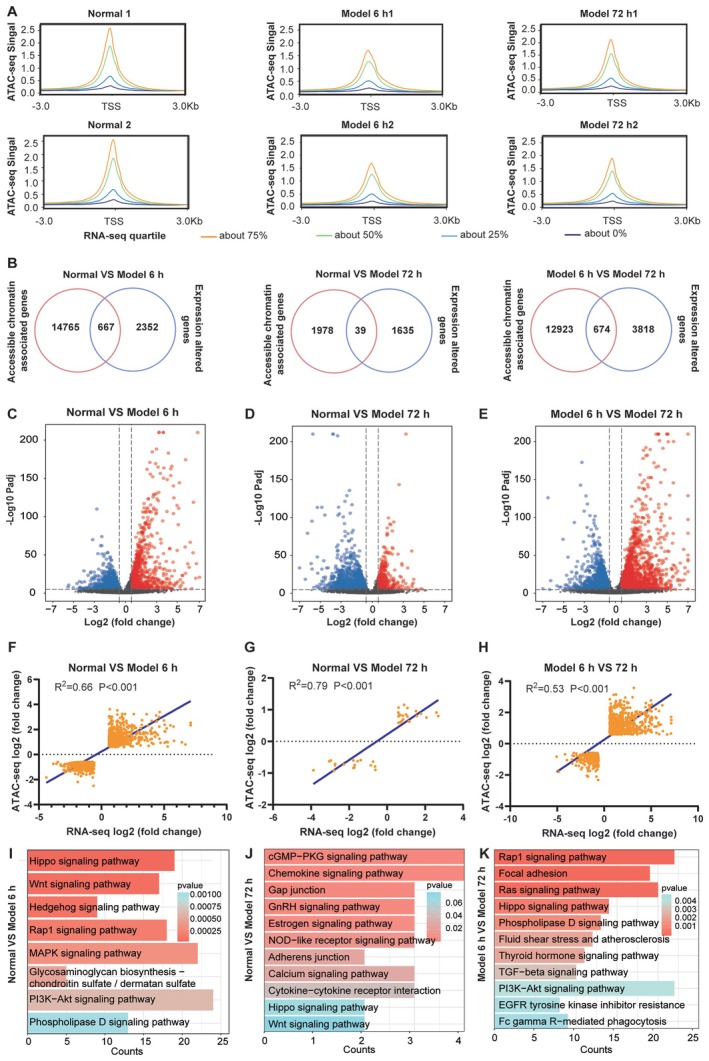
Integrative analysis of chromatin accessibility and gene expression. (A) Tetragram of ATAC‐seq signals at the TSS in the normal, 6 h model, and 72 h model groups. (B) Venn diagram showing the overlap between genes associated with regions of differential accessibility and differentially expressed genes among the normal, 6 h model, and 72 h model groups. (C–E) Volcano plot of altered genes with accessible chromatin in the normal, 6 h model, and the 72 h model groups. (F–H) Scatterplots of log2 fold changes in chromatin accessibility (ATAC‐seq) versus gene expression (RNA‐seq). (I‐K) Enriched pathways of genes with differentially accessible regions and differential expression.

We categorised RNA‐seq signals into four groups according to gene expression quartiles. Next, we analysed ATAC‐seq signals enriched in TSS regions in the form of matched RNA‐seq signals. Our results indicated that the highest quartile of gene expression correlated with increased chromatin accessibility in the 6‐h model, 72‐h model, and control group (Figure 4A). Our Venn diagram illustrated that 14,765 and 1978 genes possessed regions of differential chromatin accessibility, while 2352 and 1653 genes were differentially expressed in the 6‐h and 72‐h models relative to the control, respectively. A total of 667 genes in the 6‐h model and 39 genes in the 72‐h model exhibited both transcriptional alterations and differential chromatin accessibility compared to the control. Meanwhile, 12,923 genes showed regions of differential chromatin accessibility, and 3818 genes were differentially expressed in the 72‐h model compared to the 6‐h model. Only 674 genes exhibited both transcriptional changes and differential chromatin accessibility in the 6‐h and 72‐h models (Figure 4B), indicating that numerous genes with ATAC‐seq peaks do not undergo differential expression. This result suggests that gene expression may rely on multiple regulatory regions, many of which are located distant from the gene locus. We generated a volcano plot to display genes with altered transcription levels and regions of chromatin accessibility, and 762 genes were up‐regulated and 935 genes were down‐regulated under 6 h of TGF‐β1 treatment (Figure 4C). A total of 581 genes were up‐regulated and 794 genes were down‐regulated under 72 h of TGF‐β1 treatment (Figure 4D). Compared to the 72‐h treatment, 1269 genes were up‐regulated and 745 genes were down‐regulated under 6 h of TGF‐β1 treatment (Figure 4E). To quantitatively assess the association between chromatin accessibility and transcriptional changes, we performed linear regression analysis on log2 fold changes of ATAC‐seq and RNA‐seq data. This revealed strong positive correlations, with *R* ^2^ = 0.66 (p* < 0.0001) for the 6‐h TGF‐β1‐treated group versus controls, *R* ^2^ = 0.79 (*p* < 0.0001) for the 72‐h group versus controls, and *R* ^2^ = 0.53 (*p* < 0.0001) for the 72‐h versus 6‐h comparison (Figure 4F–H). These results indicate that chromatin remodelling dynamically correlates with transcriptional reprogramming during fibroblast‐to‐myofibroblast differentiation, with diminishing association over time, potentially reflecting stage‐specific regulatory mechanisms. Furthermore, integrating TF motif enrichment analysis (HOMER) with differentially expressed genes identified STAT3, ATF3, JUNB and FOXP1 as key regulators occupying accessible chromatin regions (q < 0.01). A regulatory network mapping these TFs to their predicted target genes (RUNX1, JUNB and SMAD6) underscores their coordinated role in driving fibrotic transcriptional programmes (Figure S1). Further KEGG analysis showed that the pathways enriched with these altered genes, including the TGF‐β, Wnt, and Hippo signalling pathways, are related to pulmonary fibrosis (Figure 4I–K).

### 3.4 Identification of Open‐Chromatin‐Bound TFs in Differentially Expressed Genes

**FIGURE 5:**
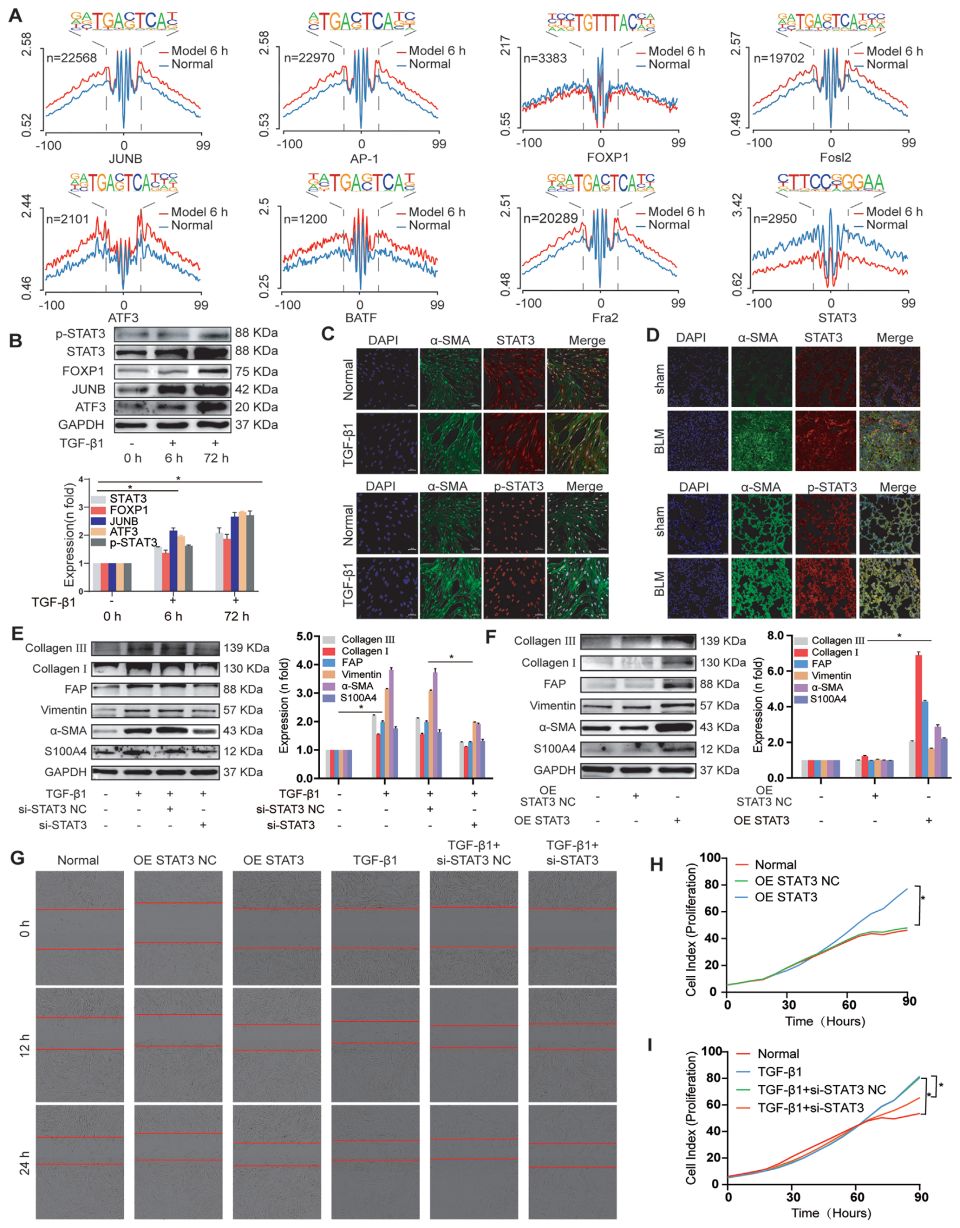
STAT3 was identified as a profibrotic TF in open chromatin regions. (A) ATAC‐seq footprinting analysis of the occupancy and accessibility of TF sites containing JUNB, BATF, FOXP1, STAT3, Fra2, AP‐1, ATF3 and FOSL2. (B) Western blot analysis revealed that the expression levels of the related TFs STAT3, p‐STAT3, FOXP1, JUNB and ATF3 were up‐regulated in TGF‐β1‐stimulated fibroblasts relative to in the control group. (C) Immunofluorescence double staining showed that the expression of p‐STAT3 and STAT3 in the TGF‐β1‐treated MRC‐5 cells were significantly higher than that in normal cells. (D) Immunofluorescence double staining showed that the expression of p‐STAT3 and STAT3 in the lung tissue of mice in the bleomycin group were significantly higher than that in normal mice. (E and F)Western blot analysis revealed that the knockdown (si) and overexpression of STAT3 (OE STAT3) down‐ or up‐regulated the expression levels of fibrosis and differentiation‐related proteins, respectively. (G) IncuCyte S3 live‐cell analysis system was employed to monitor cell migration. STAT3 overexpression (OE STAT3) promoted cell migration as TGF‐β1, and STAT3 knockdown (si) weakened cell migration. (H) IncuCyte S3 instrument detection demonstrated that the overexpression (OE STAT3) of STAT3 promoted the proliferative ability of MRC‐5 cells. (I) Compared with the normal treatment, TGF‐β1 treatment promoted cell proliferation, and STAT3 knockdown attenuated cell proliferation caused by TGF‐β1.

Accessible chromatin regions are usually essential for the binding of TFs to regulate gene expression. We annotated the presence of TF motif sequences in accessible chromatin regions on the basis of ATAC‐seq peaks. We identified 333 unique TFs in the 6‐h model group and 238 in the 72‐h model group compared to the normal group. Most of the significantly enriched TFs related to pulmonary fibrosis included STAT3, FOXP1, JUNB, ATF3, FosL2, BATF, Fra2 and AP‐1 (Figure 5A). We chose to further examine the expression level of STAT3, its phosphorylated form (p‐STAT3), FOXP1, JUNB and ATF3. Western blot analysis revealed that these TFs were up‐regulated in MRC‐5 cells following 6 and 72 h of TGF‐β1 stimulation (Figure 5B). Notably, the *p*‐value for STAT3 was among the highest in the ATAC‐seq data, indicating its significant role. Additionally, double immunofluorescence results indicated increased STAT3 and p‐STAT3 expression in myofibroblasts from MRC‐5 cells with TGF‐β1 treatment or mice with bleomycin‐induced pulmonary fibrosis, implying the critical role of STAT3 activation in driving fibroblast‐to‐myofibroblast differentiation across experimental models (Figure 5C,D). Although our previous studies indicated that STAT3 is up‐regulated during pulmonary fibrogenesis [26, 27], we did not investigate its functional and molecular mechanisms in this process. Therefore, we conducted further analysis to examine how STAT3 regulates lung fibrogenesis through both gain‐ and loss‐of‐function studies. Western blot results showed that the profibrosis‐ and differentiation‐related proteins decreased when cells were treated with STAT3‐specific siRNA (Figure 5E). In contrast, overexpression STAT3 (OE STAT3) increased the levels of profibrotic and differentiation‐related proteins, resulting in an expression pattern similar to that observed with TGF‐β1 treatment (Figure 5F). Cell proliferation and migration were monitored by using an IncuCyte S3 instrument, and the results showed that STAT3 knockdown blocked the proliferation and migration of TGF‐β1‐treated cells. Conversely, increasing STAT3 levels promoted cell proliferation and migration (Figure 5G–I). These findings suggested that STAT3 acts as a profibrotic factor that promotes the differentiation of fibroblast into myofibroblast, enhances myofibroblast proliferation and migration, and increases collagen deposition.

### 3.5 STAT3 Facilitated the Formation of SEs to Promote the Expression of Target Genes

**FIGURE 6:**
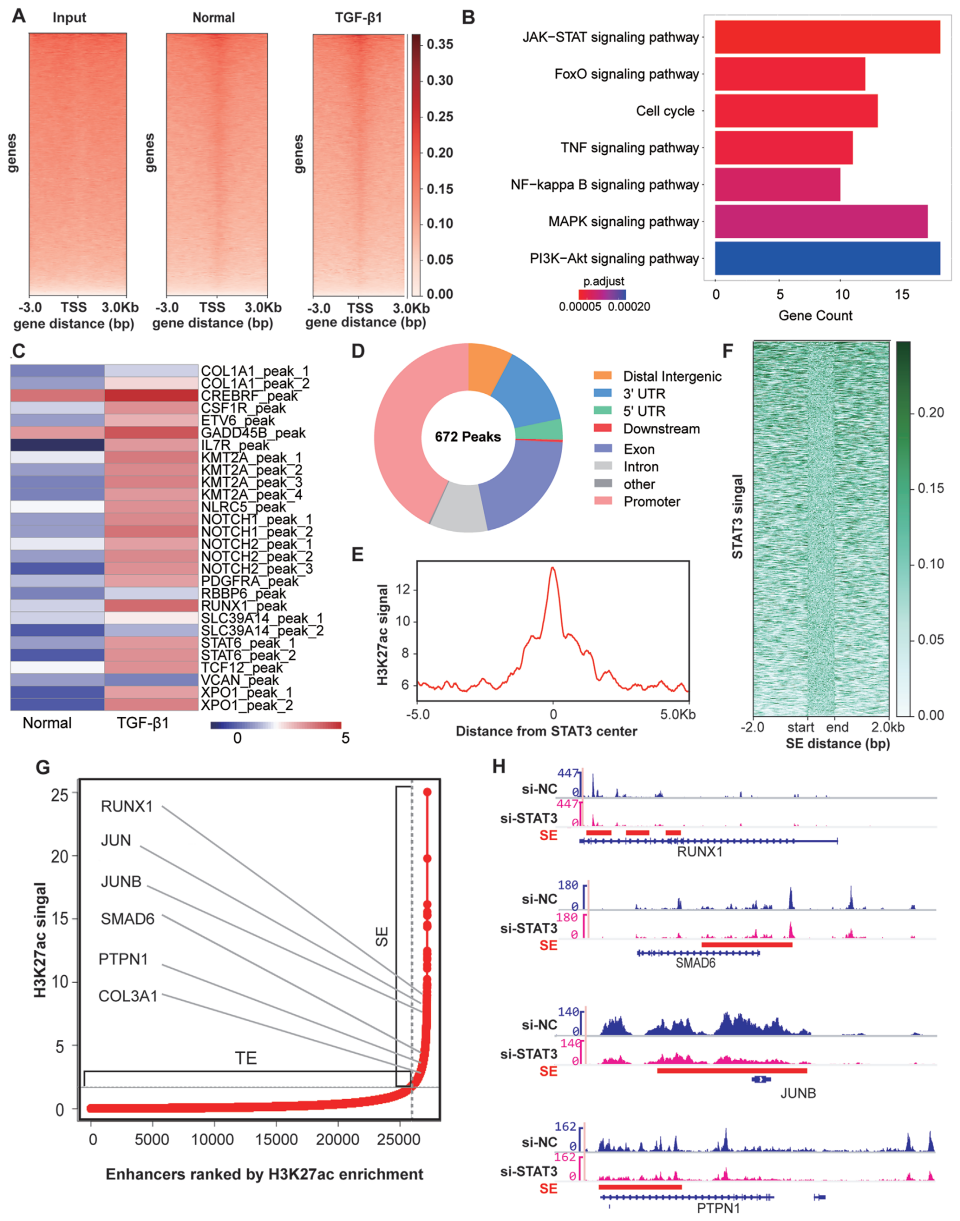
Analysis of the relationship of STAT3 and gene expression. (A) Heatmaps of the normalised ChIP‐seq signals of STAT3 with respect to the TSS. (B) Pathways of STAT3‐associated genes enriched in KEGG analysis. (C) Cluster plot showing accessible chromatin regions that were highly enriched in STAT3. (D) Pie chart showing the distribution of STAT3 ChIP‐seq peaks at genomic loci. (E) Line graphs presenting the occupancy signals of H3K27ac near the centre of STAT3 binding peaks. (F) Heatmap of STAT3 signals at specific SE loci. (G) Number of SEs defined by ranking H3K27ac binding signals. Geometric inflection points are indicated by a dashed line. Typical enhancers and SEs are indicated on the left and right of the dashed line, respectively. Representative STAT3‐bound SE‐associated genes are labelled. (H) H3K27ac ChIP‐seq signal tracks (normalised read counts, Y‐axis) at SE regions of RUNX1, SMAD6, JUNB and PTPN1 in control (si‐NC) and STAT3‐knockdown (si‐STAT3) MRC‐5 cells. Red lines highlight SE regions defined by H3K27ac aggregation.

We next examined the genome‐wide binding of STAT3 to identify its target genes using chromatin immunoprecipitation and high‐throughput ChIP‐seq analysis in TGF‐β1‐treated cells. STAT3 binding to chromatin increased during fibroblast‐to‐myofibroblast differentiation (Figure 6A). KEGG pathway analysis indicated that genes with specific binding sequences for STAT3 were significantly enriched in several pathways, including the JAK–STAT signalling pathway, FoxO signalling pathway, and cell cycle. This suggests that STAT3 plays a role in cell proliferation and differentiation (Figure 6B). Additionally, we combined STAT3 ChIP‐seq, ATAC‐seq, and RNA‐seq analyses to explore the relationships between STAT3 enrichment, chromatin accessibility, and gene transcription. The results revealed that in TGF‐β1‐treated cells, STAT3 was highly enriched in regions of accessible chromatin (Figure 6C). Moreover, we found that differential STAT3 binding sites were enriched in promoters and distal regions away from transcription start sites, such as introns and intergenic regions (Figure 6D). The high intensity of H3K27ac modification is an indicator that distinguishes SEs from typical enhancers [28, 29, 30]. Therefore, we performed ChIP‐seq on H3K27ac in TGF‐β1‐stimulated MRC‐5 cells. H3K27ac with active histone modifications was enriched at STAT3‐bound loci, indicating that STAT3 might be a SE‐associated factor (Figure 6E). The global heatmap showed that STAT3 was enriched at super enhancers (SEs) (Figure 6F). Using the ROSE program, we identified 580 SEs and 1282 SE‐associated genes, 76 of which were identified as potential STAT3 targets. These genes included the fibrosis‐associated genes RUNX1, JUNB, JUN, SMAD6, PTPN1 and COL3A1 (Figure 6G). By using IGV, we further demonstrated that STAT3 exhibited strong co‐occupancy with H3K27ac in the SE region of genes, including RUNX1, JUNB, SMAD6 and PTPN1. To clearly demonstrate the importance of STAT3 in SE formation, we conducted H3K27ac ChIP‐seq on MRC‐5 cells with STAT3 knocked down (si‐STAT3). The analysis revealed a marked reduction in H3K27ac enrichment at SE regions of RUNX1, SMAD6, JUNB and PTPN1 following STAT3 depletion (Figure 6H). These results confirm that STAT3 is essential for preserving H3K27ac modifications and the integrity of SEs. This finding further supports its role as an epigenetic driver of fibrotic gene expression.

**FIGURE 7:**
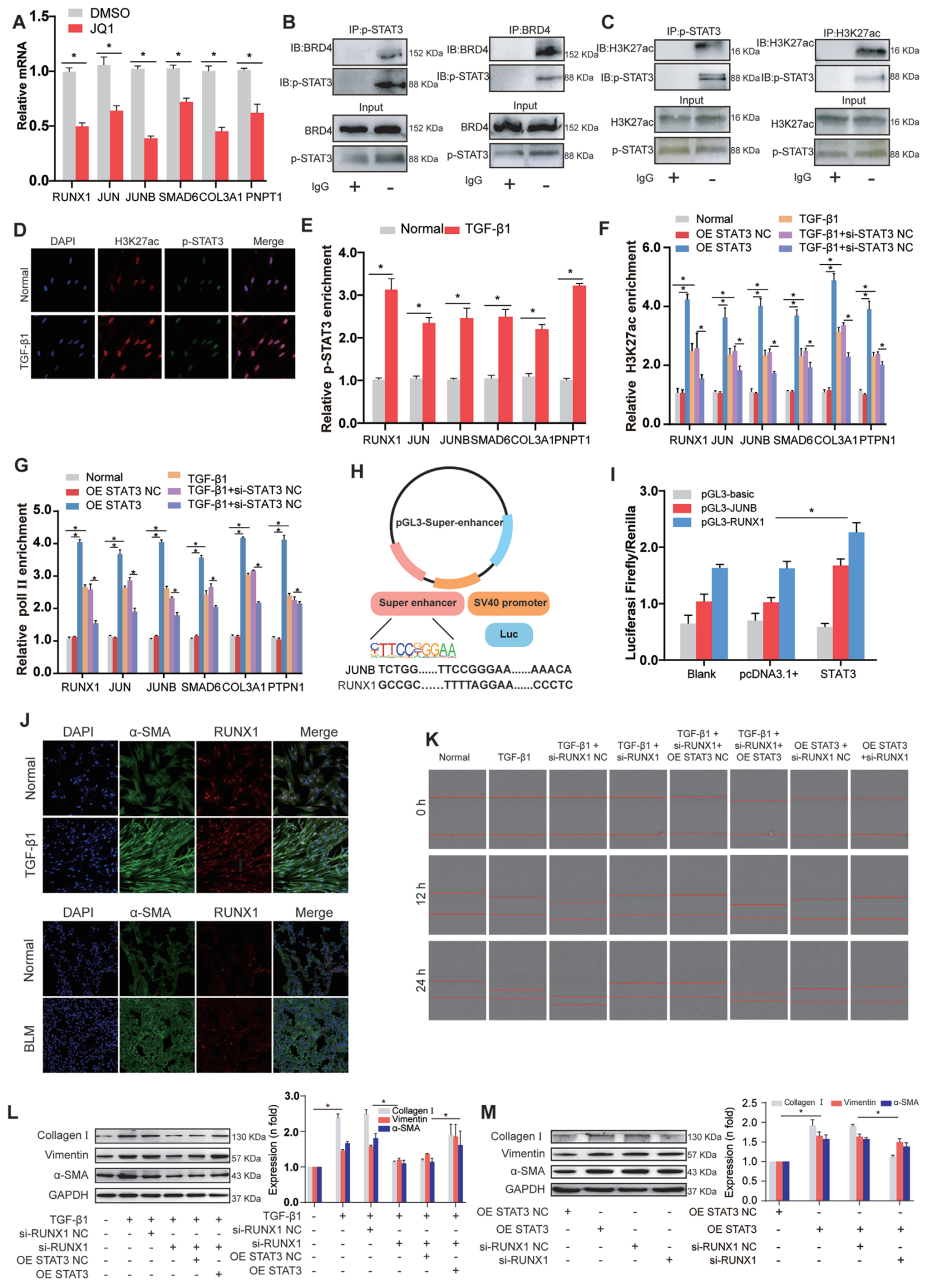
STAT3 promoted target gene expression by contributing to SE formation. (A) mRNA levels of target genes with or without 24 h of JQ1 treatment were analysed. JQ1 inhibited the expression of SE‐related target genes, such as RUNX1, JUNB, JUN, SMAD6, COL3A1 and PTPN1. (B) Co‐IP analysis of endogenous interaction between BRD4 and p‐STAT3. Cell lysates were immunoprecipitated with anti‐BRD4 and anti‐p‐STAT3 antibodies, followed by immunoblotting with indicated antibodies. IgG serves as negative control. (C) Co‐immunoprecipitation (Co‐IP) analysis of p‐STAT3 and H3K27ac interactions. Left panel: Cell lysates from TGF‐β1‐treated MRC‐5 cells were immunoprecipitated with anti‐p‐STAT3 or IgG antibodies, followed by Western blotting for p‐STAT3 and H3K27ac. Right panel: Reciprocal Co‐IP using anti‐H3K27ac antibodies, blotted for H3K27ac and p‐STAT3. Input lanes represent total protein levels. (D) Double immunofluorescence staining confirmed that H3K27ac (red) and p‐STAT3 (green) were highly co‐expressed and co‐located in the TGF‐β1 group relative to the control group. (E) CUT&RUN‐qPCR analysis demonstrated that TGF‐β1 promoted p‐STAT3 enrichment in the SE regions of target genes, including RUNX1, JUNB, JUN, SMAD6, COL3A1 and PTPN1. (F) CUT&RUN‐qPCR analysis demonstrated that STAT3 overexpression (OE STAT3) promoted H3K27ac modification at STAT3‐bound loci, whereas STAT3 knockdown (si) weakened H3K27ac modification. (G) Pol II was enriched in the SEs of RUNX1, JUNB, JUN, SMAD6, COL3A1 and PTPN1 in the STAT3 overexpression (OE STAT3) group, whereas Pol II enrichment was attenuated in the STAT3 knockdown (si) group. (H) Schematic of pGL3‐SE vectors. (I) Detection of luciferase activity demonstrated that STAT3 enhanced pGL3‐SE activities to promote the gene transcription of RUNX1 and JUNB. (J) Immunofluorescence double staining showed that the expression of α‐SMA and RUNX1 was significantly higher in MRC‐5 cells treated with TGF‐β1 and the lung tissue of bleomycin‐induced mice than in the control group. BLM indicates bleomycin. (K) Migration images were automatically monitored using the IncuCyte S3 Live‐Cell Analysis System. Compared with the TGF‐β1 group, the RUNX1 knockdown (si) repressed myofibroblast migration at different time points, and STAT3 overexpression (OE STAT3) reversed the decreased migration ability of myofibroblasts induced by si‐RUNX1. (L) Western blot showed that si‐RUNX1 decreased the levels of fibrotic proteins, and STAT3 overexpression (OE STAT3) reversed the decreased levels of fibrotic proteins induced by si‐RUNX1, including α‐SMA, vimentin and collagen I. (M) Rescue experiments indicated that si‐RUNX1 reversed the increased trends of collagen I, vimentin, and α‐SMA levels caused by overexpressed STAT3.

Bromodomain‐containing protein 4 (BRD4) is a key functional protein in the bromodomain and extraterminal domain family. It recruits transcriptional regulatory complexes that acetylate lysine residues on histone tails, thereby influencing gene expression [31, 32, 33]. Research indicates that BRD4 is significantly enriched in SEs, which drive gene expression [34, 35]. To confirm the role of SE formation in target gene regulation, the BRD4 inhibitor JQ1 was applied. The results showed that JQ1 significantly reduced RNA levels of RUNX1, JUNB, JUN, SMAD6, PTPN1 and COL3A1, confirming their reliance on BRD4‐mediated SE assembly (Figure 7A). Co‐immunoprecipitation assays revealed a physical interaction between BRD4 and phosphorylated STAT3 (p‐STAT3) (Figure 7B), suggesting their cooperative role in chromatin remodelling. Additionally, co‐immunoprecipitation assays showed bidirectional interactions between H3K27ac and p‐STAT3 (Figure 7C). Moreover, TGF‐β1 increased the co‐location of these proteins, indicating that STAT3 may be involved in SE formation (Figure 7D). We designed primers for the STAT3 peak of target genes and used anti‐STAT3 or anti‐H3K27ac antibodies to enrich DNA fragments to determine whether the STAT3 binding region is enriched with activating H3K27ac modifications. CUT&RUN‐qPCR showed that TGF‐β1 increased STAT3 enrichment in the SEs of its target genes, including RUNX1, JUNB, JUN, SMAD6, PTPN1 and COL3A1 (Figure 7E). We then compared the occupancy of H3K27ac at the STAT3‐bound loci of these genes through STAT3 overexpression and knockdown in TGF‐β1 induced MRC‐5 cells. The results showed that STAT3 promoted the enrichment of H3K27ac in RUNX1, JUNB, JUN, SMAD6, PTPN1 and COL3A1 genes (Figure 7F). We transfected an overexpression vector for STAT3 into MRC‐5 cells to further substantiate the transcriptional regulatory role on target genes and determine whether STAT3 can induce the enrichment of RNA polymerase II (Pol II) at the promoters. Similar to TGF‐β1 treatment, STAT3 overexpression also increased the enrichment of Pol II in genes, such as RUNX1, JUNB, JUN, SMAD6, PTPN1 and COL3A1. Conversely, in TGF‐β1‐treated cells, STAT3 knockdown reduced Pol II levels (Figure 7G). Next, we inserted the SE element of genes (RUNX1 and JUNB) into the pGL3‐promoter vector (named pGL3‐SE) (Figure 7H). The result of the dual‐luciferase reporter system assay indicated that pGL3‐SEs promoted the transcription levels of RUNX1 and JUNB under STAT3 treatment (Figure 7I). RUNX1 was selected for further exploration of the regulatory role of STAT3. Double immunofluorescence results showed that TGF‐β1 or bleomycin increased the expression of RUNX1 and α‐SMA in MRC‐5 cells or mice lung tissue, suggesting that RUNX1 promotes the occurrence of pulmonary fibrosis (Figure 7J). The role of RUNX1 in pulmonary fibrosis was elucidated by examining myofibroblast migration and collagen deposition. We monitored cell migration with the IncuCyte S3 instrument. The results indicated that silencing RUNX1 inhibited the migration of cells treated with TGF‐β1. Additionally, the results from the rescue experiment revealed that overexpressing STAT3 reversed the reduction in cell migration induced by RUNX1 knockdown (Figure 7K). Treating cells with specific siRNA targeting RUNX1 resulted in a reduction of profibrotic and differentiation‐related proteins. The rescue assay result showed that overexpression of STAT3 levels reversed the reduction of fibrosis‐related protein levels caused by RUNX1 knockdown (Figure 7L). Further rescue experiment also showed that si‐RUNX1 reversed the increase in collagen I, vimentin, and α‐SMA levels caused by overexpressed STAT3 (Figure 7M), suggesting that RUNX1 may directly contribute to fibrotic matrix accumulation during disease progression.

To confirm the collaborative functions of STAT3‐SE‐regulated genes, including JUNB and SMAD6, we conducted functional studies. Immunofluorescence co‐staining showed that JUNB and SMAD6 co‐localised with α‐SMA positive myofibroblasts in both TGF‐β1‐treated MRC‐5 cells and bleomycin‐induced fibrotic lung tissues (Figure S2A,B). Knockdown of SMAD6 or JUNB using siRNA significantly reduced the migration and proliferation of myofibroblasts, while STAT3 overexpression (OE STAT3) reversed this effect (Figure S2C,D). Additionally, knockdown of SMAD6 with siRNA significantly decreased the levels of α‐SMA, vimentin, and collagen I in TGF‐β1‐treated MRC‐5 cells (Figure S2E). Conversely, overexpression of STAT3 (OE STAT3) partially rescued the inhibitory effects of si‐SMAD6 on fibrotic protein expression (Figure S2F). Similarly, JUNB knockdown reduced collagen deposition and myofibroblast migration, while STAT3 overexpression restored these phenotypes (Figure S2G,H). Overall, these results show that STAT3‐SE coordinates a network involving RUNX1, JUNB, and SMAD6 to promote the differentiation of fibroblasts into myofibroblasts.

## 4 Discussion

The transformation of fibroblasts into myofibroblasts is a key feature of IPF [5, 36, 37]. However, the epigenetic mechanisms driving this process remain incompletely understood. Our study combines multi‐omics analyses to identify STAT3 as a key regulator of super enhancer (SE) assembly. This process connects chromatin accessibility dynamics with the transcriptional activation of fibrotic genes. By utilising ATAC‐seq, RNA‐seq and ChIP‐seq, we show that STAT3 regulates SE formation via H3K27ac modification, promoting the expression of RUNX1, JUNB, SMAD6, and other genes associated with fibrosis. This research broadens our understanding of STAT3's functions beyond its traditional role in TGF‐β signalling and introduces a new epigenetic pathway that could have therapeutic applications for treating fibrotic diseases.

STAT3 is well‐known for its activation in fibrosis and cancer, which usually occurs through JAK–STAT or TGF‐β/SMAD signalling pathways [38, 39]. However, our findings reveal a distinct mechanism in the pathogenesis of IPF. In cancer progression, STAT3 mainly promotes oncogenic transcription through well‐established phosphorylation‐dependent pathways, such as the JAK/STAT3 or IL‐6/STAT3 axes, which upregulate cyclin D1, Bcl‐xL and survivin [40]. In contrast, in pulmonary fibrosis, STAT3 directly recruits H3K27ac‐modified histones to super enhancer (SE) loci (Figure 7C,D). This chromatin remodelling function demonstrates STAT3's adaptability as an epigenetic regulator that varies with context. Importantly, our ChIP‐seq analysis of H3K27ac in cells with STAT3 knockdown (Figure 6H) confirmed that STAT3 is essential for maintaining super enhancer integrity, as its loss significantly decreased H3K27ac enrichment at fibrogenic loci.

We discovered that phosphorylated STAT3 (p‐STAT3) physically interacts with BRD4 (Figure 7B), offering important insights into the assembly of super enhancers (SEs). BRD4 is a bromodomain protein that is abundant at super enhancers (SEs). It helps with transcriptional elongation by recruiting mediator complexes [21]. Although BRD4 inhibitors such as JQ1 broadly disrupt transcription driven by super enhancers (SEs) [41], our findings indicate that STAT3 serves as a scaffold, recruiting BRD4 to fibrogenic SEs and facilitating localised histone acetylation (Figure 7A,B). This model aligns with the observed rescue of JUNB and SMAD6 expression by STAT3 overexpression (Figure S2). The co‐immunoprecipitation of p‐STAT3 and H3K27ac (Figure 7C) supports a cooperative mechanism where STAT3 stabilises BRD4‐mediated acetylation, establishing a feedback loop that enhances fibrotic transcription. This precise targeting of super enhancers (SEs) differs from the effects of broad‐spectrum SE inhibitors, emphasising STAT3's crucial role in potential therapeutic applications.

This study makes a significant advancement by identifying a gene network regulated by STAT3‐SE that includes RUNX1, JUNB and SMAD6. Previous studies concentrated on individual fibrotic pathways. In contrast, our functional validations show that STAT3 coordinates these genes synergistically. Knockdown of SMAD6 or JUNB reduced myofibroblast migration and collagen deposition, effects that were restored by STAT3 overexpression (Figure S2C–H). In addition, silencing RUNX1 reversed fibrosis driven by STAT3 (Figure 7M), further confirming the interdependency within this network. By integrating TF motif analysis with differentially expressed genes (Figure 4F–H), we map a regulatory hierarchy. In this hierarchy, STAT3 enhances chromatin accessibility, allowing AP‐1 and JUNB to boost fibrotic signals.

Current antifibrotic strategies that target TGF‐β encounter challenges from the compensatory activation of parallel signalling pathways, such as Wnt/β‐catenin and PDGFR. These pathways sustain fibroblast activation and lead to dose‐limiting toxicities because of TGF‐β's diverse roles in immune regulation and tissue repair [25, 41]. We identified STAT3 as a context‐dependent epigenetic scaffold, which changes how we approach therapeutic targeting. Instead of broadly suppressing enhancer clusters with BET bromodomain inhibitors like JQ1, we can selectively disrupt STAT3‐mediated enhancer phase separation using approaches like dCas9‐KRAB silencing. This method allows us to inactivate harmful super enhancers while maintaining normal enhancer structures. There is a strong correlation between the fold changes observed in ATAC‐seq and RNA‐seq (*R* ^2^ = 0.53–0.79, Figure 4F–H). This correlation highlights chromatin remodelling as a key driver of transcriptional reprogramming.

Although our work identifies STAT3‐SE as a key mechanism, several questions still exist. First, exploring the tissue‐specific characteristics of SE landscapes is important, as lung fibroblasts may have unique epigenetic vulnerabilities compared to liver or heart fibroblasts. Second, the role of non‐coding RNAs in influencing STAT3‐SE activity, similar to chromosome‐associated regulatory RNAs (carRNAs) in METTL3‐regulated chromatin, deserves further investigation [42]. Finally, validating STAT3‐targeted epigenetic modulators in animal models is crucial for translating these findings into effective therapies.

By defining the STAT3‐SE axis as a key driver of fibrogenic transcription, this study shifts the therapeutic approach for IPF from inhibiting pathways to targeting specific enhancers. Our multi‐omics framework not only elucidates the epigenetic basis of fibroblast activation but also provides a blueprint for exploiting context‐dependent vulnerabilities in fibrotic diseases. Future efforts to dissect tissue‐specific SE architectures and evaluate STAT3‐directed therapies could unlock transformative treatments for IPF and other diseases.

## Author Contributions

**Yujie Wang:** conceptualization (equal), data curation (equal), methodology (equal), software (equal), writing – original draft (equal). **Yaqin Zhao:** data curation (equal), formal analysis (equal), software (equal), writing – original draft (equal). **Guohong Cao:** formal analysis (equal), resources (equal), supervision (equal). **Mengqi Jiang:** data curation (equal), investigation (equal), methodology (equal). **Xinglong Yuan:** resources (equal), validation (equal). **Hongbo Li:** data curation (equal), investigation (equal), visualization (equal), writing – review and editing (equal). **Xiaodong Song:** conceptualization (equal), funding acquisition (equal), resources (equal), visualization (equal), writing – review and editing (equal). **Jinjin Zhang:** conceptualization (equal), data curation (equal), validation (equal), writing – review and editing (equal). **Changjun Lv:** data curation (equal), funding acquisition (equal), validation (equal), writing – review and editing (equal). **Songzi Zhang:** data curation (equal), formal analysis (equal), methodology (equal), writing – review and editing (equal).

## Conflicts of Interest

The authors declare no conflicts of interest.

## Supporting information


**Figure S1.** Regulatory network integrating transcription factor motifs and target genes in open chromatin regions. Regulatory network integrating transcription factor (TF) motifs enriched in open chromatin regions (HOMER analysis, q < 0.01) and their predicted target genes (differentially expressed genes, FDR < 0.05). Motif analysis using HOMER identified STAT3, ATF3, JUNB and FOXP1 as top enriched TFs in open chromatin regions. A regulatory network was constructed using Cytoscape, linking TF motifs to their predicted target genes (e.g., RUNX1, JUNB, SMAD6), demonstrating coordinated chromatin accessibility and transcriptional activation.
**Figure S2**. SMAD6 and JUNB co‐localization with α‐SMA and knockdown effects on myofibroblast migration and fibrotic protein expression. (A, B) Double immunofluorescence staining of SMAD6 or JUNB (red) and α‐SMA (green) in TGF‐β1‐treated MRC‐5 cells and bleomycin (BLM)‐induced fibrotic lung tissues. Nuclei were stained with DAPI (blue). (C, D) Scratch wound healing assay showing reduced migration in si‐JUNB/si‐SMAD6‐treated MRC‐5 cells. Wound healing assay showing restored migration capacity in OE STAT3 rescued cells. (E, F) Western blot showed that si‐SMAD6 decreased the levels of fibrotic proteins, and STAT3 overexpression (OE STAT3) reversed the decreased the levels of fibrotic proteins induced by si‐SMAD6, including α‐SMA, vimentin and collagen I. (G, H) Western blot showed that si‐JUNB decreased the levels of fibrotic proteins, and STAT3 overexpression (OE STAT3) reversed the decreased the levels of fibrotic proteins induced by si‐JUNB, including α‐SMA, vimentin and collagen I.

## Data Availability

The authors declare that the data that support the findings of this study are available from the corresponding author upon reasonable request.
