# Supplementary material for: STAT3 Facilitates Super Enhancer Formation to Promote Fibroblast‐To‐Myofibroblast Differentiation by the Analysis of ATAC‐Seq, RNA‐Seq and ChIP‐Seq
Source: J Cell Mol Med. 2025 Jun 4;29(11):e70639. doi: 10.1111/jcmm.70639 (PMC12135348; doi:10.1111/jcmm.70639)
Supplement: Supplementary file 1 — Figure S1. Regulatory network integrating transcription factor motifs and target genes in open chromatin regions. Regulatory network integrating transcription factor (TF) motifs enriched in open chromatin regions (HOMER analysis, q < 0.01) and their predicted target genes (differentially expressed genes, FDR < 0.05). Motif analysis using HOMER identified STAT3, ATF3, JUNB and FOXP1 as top enriched TFs in open chromatin regions. A regulatory network was constructed using Cytoscape, linking TF motifs to their predicted target genes (e.g., RUNX1, JUNB, SMAD6), demonstrating coordinated chromatin accessibility and transcriptional activation. Figure S2. SMAD6 and JUNB co‐localization with α‐SMA and knockdown effects on myofibroblast migration and fibrotic protein expression. (A, B) Double immunofluorescence staining of SMAD6 or JUNB (red) and α‐SMA (green) in TGF‐β1‐treated MRC‐5 cells and bleomycin (BLM)‐induced fibrotic lung tissues. Nuclei were stained with DAPI (blue). (C, D) Scratch wound healing assay showing reduced migration in si‐JUNB/si‐SMAD6‐treated MRC‐5 cells. Wound healing assay showing restored migration capacity in OE STAT3 rescued cells. (E, F) Western blot showed that si‐SMAD6 decreased the levels of fibrotic proteins, and STAT3 overexpression (OE STAT3) reversed the decreased the levels of fibrotic proteins induced by si‐SMAD6, including α‐SMA, vimentin and collagen I. (G, H) Western blot showed that si‐JUNB decreased the levels of fibrotic proteins, and STAT3 overexpression (OE STAT3) reversed the decreased the levels of fibrotic proteins induced by si‐JUNB, including α‐SMA, vimentin and collagen I. [file JCMM-29-e70639-s001.docx]

~~
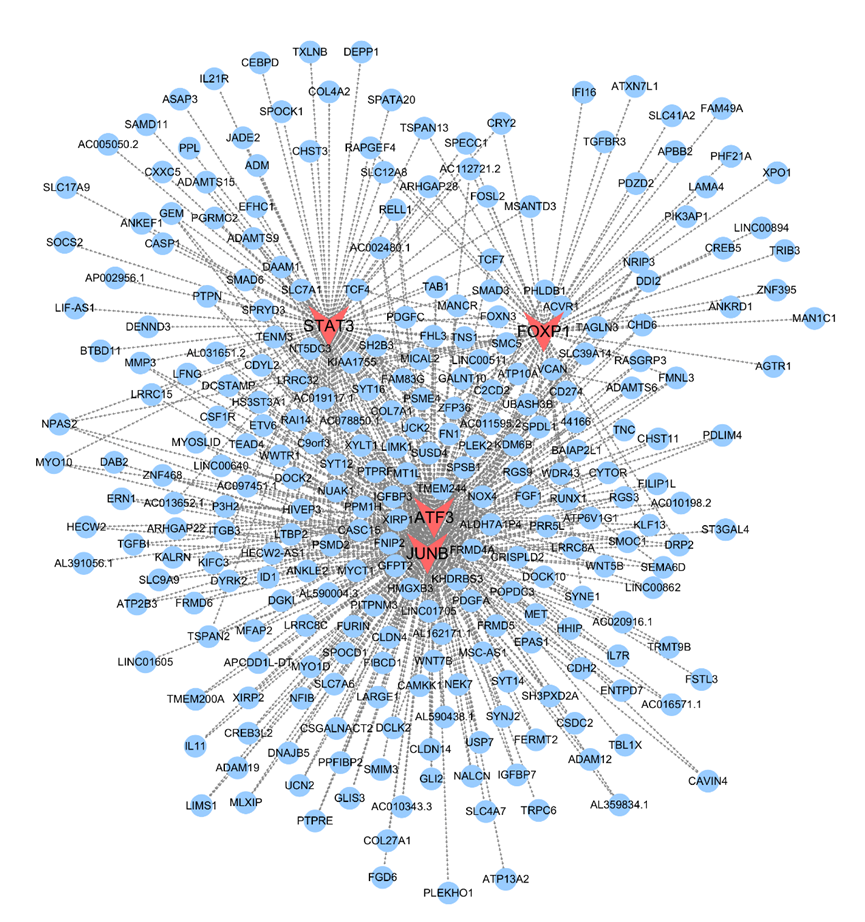
~~**Supplementary Fig 1. Regulatory network integrating transcription factor motifs and target genes in open chromatin regions.**Regulatory network integrating transcription factor (TF) motifs enriched in open chromatin regions (HOMER analysis, q < 0.01) and their predicted target genes (differentially expressed genes, FDR < 0.05). Motif analysis using HOMER identified STAT3, ATF3, JUNB, and FOXP1 as top enriched TFs in open chromatin regions. A regulatory network was constructed using Cytoscape, linking TF motifs to their predicted target genes (e.g., RUNX1, JUNB, SMAD6), demonstrating coordinated chromatin accessibility and transcriptional activation.


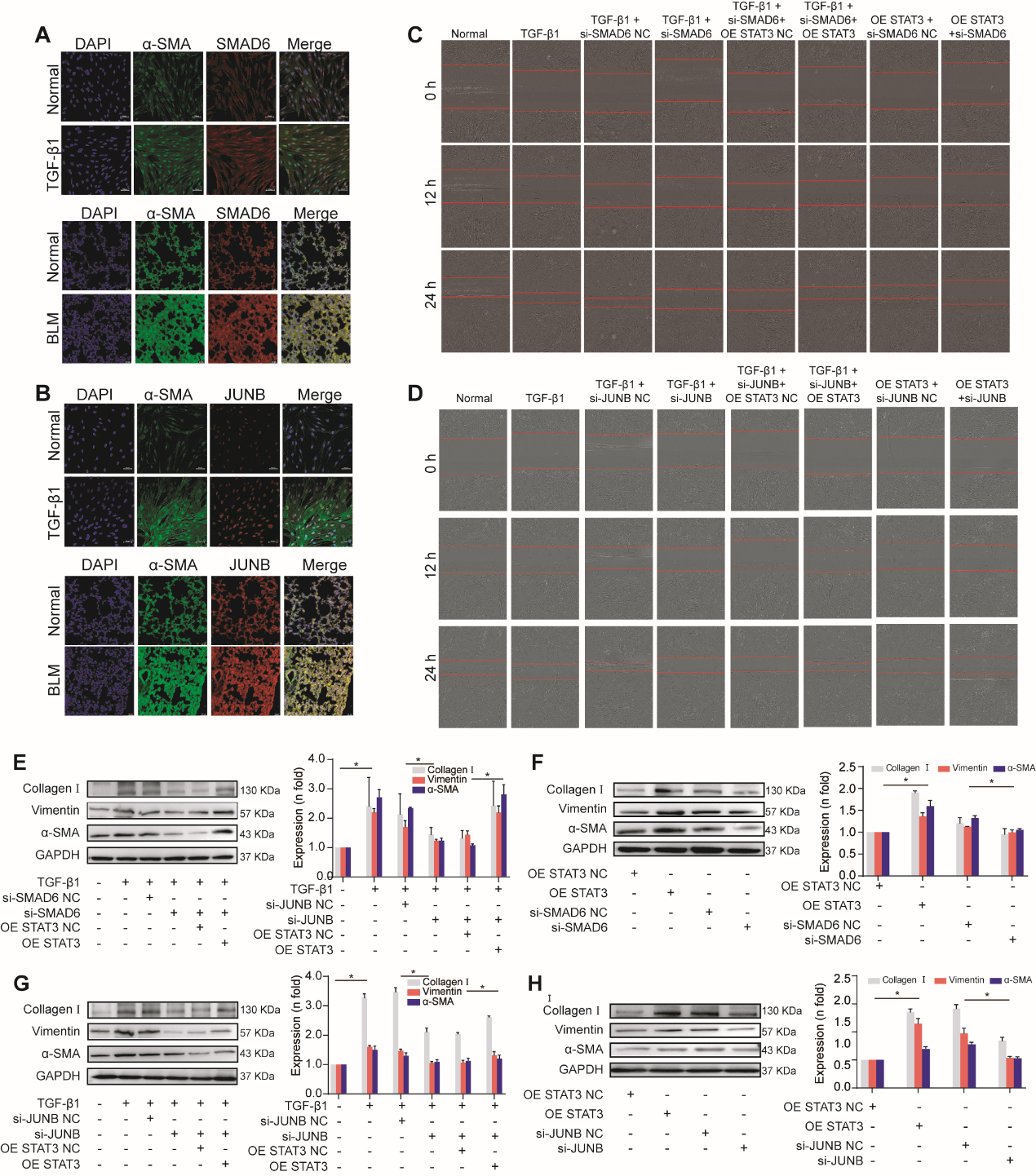
**Supplementary Fig 2. SMAD6 and JUNB co-localization with α-SMA and knockdown effects on myofibroblast migration and fibrotic protein expression.**
 (A, B) Double immunofluorescence staining of SMAD6 or JUNB (red) and α-SMA (green) in TGF-β1-treated MRC-5 cells and bleomycin (BLM)-induced fibrotic lung tissues. Nuclei were stained with DAPI (blue). (C, D) Scratch wound healing assay showing reduced migration in si-JUNB/si-SMAD6-treated MRC-5 cells. Wound healing assay showing restored migration capacity in OE STAT3 rescued cells. (E, F) Western blot showed that si-SMAD6 decreased the levels of fibrotic proteins, and STAT3 overexpression (OE STAT3) reversed the decreased the levels of fibrotic proteins induced by si-SMAD6, including α-SMA, vimentin, and collagen I. (G, H) Western blot showed that si-JUNB decreased the levels of fibrotic proteins, and STAT3 overexpression (OE STAT3) reversed the decreased the levels of fibrotic proteins induced by si-JUNB, including α-SMA, vimentin, and collagen I.
